# Effect of preservation on the mechanical properties of 3D printing resins for occlusal splints

**DOI:** 10.1590/1807-3107bor-2025.vol39.060

**Published:** 2025-06-02

**Authors:** Betty Salazar MAYTA, Laís Alves CANDIDO, Karla ZANCOPÉ, Paulo Cézar SIMAMOTO, Gustavo Mendonça, Flávio Domingues das Neves

**Affiliations:** (a) Universidade Federal de Uberlândia – UFU, Scholl of Dentistry, Uberlândia, MG, Brazil.; (b) Universidade Federal de Uberlândia – UFU, Scholl of Dentistry, Department of Occlusion, Fixed Prosthesis and Dental Materials, Uberlândia, MG, Brazil.; (c) Virginia Commonwealth University, Scholl of Dentistry, Department of General Practice, Richmond, Virginia, USA.

**Keywords:** Occlusal Splints, computer-Aided Design, Bruxism, Mechanical Tests, Immersion

## Abstract

This study aims to evaluate the mechanical properties of 3D printing resins for occlusal splints (OS) under different preservation conditions based on sleep duration. The study factors (n = 10) included material types: PriZma 3D Bio Splint (PZ), Cosmos Splint (CS), NightGuard Firm (NGF), and self-polymerizing acrylic resin (control group - CG); preservation: artificial saliva (S), natural water (W), 0.12% chlorhexidine (C), and Corega tabs (CTs); and evaluation times: T0 (24 h after fabrication) and 30 d after exposure. Further, the surface roughness (Ra), Knoop microhardness (KH), three-point flexural strength (FS), and flexural modulus (FM) were analyzed. In terms of the time and material, Tukey’s test revealed no significant differences in Ra between the groups at 24 h. After 30 d, PZ showed significantly lower values than the other groups (p < 0.05). For the KH, NGF was significantly higher than that in all groups at 24 h (p < 0.05); after 30 d, the NGF decreased in W and C (p < 0.05), and the NGF and PZ exhibited elevated values in S and CT, respectively. The Kruskal–Wallis test and multiple comparisons showed that the PZ had a higher value at 24 h (p < 0.05). Post 30 d, all 3D-printing resins increased values in CT and S (p < 0.05), and the PZ decreased in the C and W (P < 0.05). Finally, the FM increased under all conditions. The preservation of S and CT partially enhanced the mechanical properties of the OS.

## Introduction

An occlusal splint (OS) is widely used in clinical practice as a complementary alternative in the treatment of bruxism^1^ and the consequent temporomandibular joint disorders (TMJDs).^2,3^ In the former, it serves as protection against tooth wear or extensive fractures,^3,4^ whereas in the latter, it aids in the musculoskeletal realignment, which enables the condyles to find an orthopedically stable position, which is the most comfortable one provided by even and simultaneous contacts. Their use is recommended during sleep bruxism (SB) because it involves rhythmic (phasic) or non-rhythmic (tonic) chewing muscle activity. Emphasizing that SB is not a movement or sleep disorder in healthy individuals and is centrally regulated is important.^5,6^ OS can alleviate 70–90% of TMJD symptoms with a low risk of side effects when they are properly prescribed and fabricated.^7,8,9,10^

One fabrication method of OS is computer-aided design/computer-aided manufacturing (CAD/CAM).^3,11,12^ In this approach, additive manufacturing is widely adopted because of its shorter fabrication time, better material utilization, and ease of obtaining digital impressions.^11,13-15^ The use of 3D printing resins is required, which possess the quality of being “liquid,”^13^ and their chemical composition differs from conventional ones.^16^ Factors such as layer thickness, laser power, post-curing time, etc., affect the physical and mechanical properties of the material, which impact its longevity.^11,15^ Therefore, ensuring proper retention and stability is crucial for withstanding significant occlusal forces of up to 785 N without undergoing alterations.^6,9,10,12,17^

The ideal properties of an OS include low roughness, high hardness, and high fracture resistance. Roughness, which is affected by the polishing process, can affect biofilm formation by bacterial adhesion. Surface imperfections, detectable by the sensitive tongue, can hinder continuous use and treatment adherence.^11,16^ Hardness is essential to resist loads and prevent permanent deformation; therefore, it is advisable to opt for materials with high hardness.^16^ Flexural strength (FS) and flexural modulus (FM) are crucial parameters that determine the ability of the material to resist deformation until fracture under a specific load. These properties are directly related to both the type of resin used and the thickness of the OS.^18^

The preservation, hygiene, and disinfection of OS after installation are important to maintain structural integrity, increasing longevity, and promoting good oral health.^19^ Different preservations have varying effects on mechanical properties. For example, prolonged exposure to water can cause the OS to expand because of sorption, thereby affecting its hardness, strength, and FM by plasticizing the polymer network.^10,19^ Chlorhexidine, which is commonly used in dentistry as an antiseptic, possesses broad-spectrum antimicrobial properties. However, constant exposure can cause discoloration, thereby altering the appearance of the device and affecting the mechanical properties of acrylic resins.^20^

Effervescent tablets based on alkaline peroxides are another option in the market, which offer a direct chemical method without dosing requirements. These tablets dissolve in water, generating effervescence that produces oxygen-free radicals with antimicrobial action and enzymes that break down biofilm proteins.^19^Dry storage conditions can increase surface roughness (Ra) and FS values.

Considering these aspects and the widespread use of OS fabrication using the CAD/CAM system, understanding the factors that impact its use and the benefits it provides to patients is necessary. Therefore, this study aims to evaluate the effect of the preservation method on the Ra, Knoop microhardness (KH), FS, and FM of the 3D printing resins used for OS. The null hypothesis was that the mechanical properties of the OS are not affected by the preservation method.

## Methods

Three 3D printing resins were used: Prizma Bio Splint (PZ) (Makerteach Labs, Tatuí, Brazil); Cosmos Splint (CS) (Yller Biomaterials, Pelotas, Brazil); and NightGuard Firm (NGF) (SprintRay Inc., Los Angeles, USA). A control group of self-curing acrylic resin (CG) (Auto Resin TDV, Santa Catarina, Brazil) was also used. A research randomizer was used to randomize the samples. Additional details are presented in Table 1.

**Table 1:** Material composition information provided by the manufacturer.

Material	Type	Shade	Manufacturer	Composition
Prizma 3D Bio Splint	3D printing resin	Transparent	Makerteach Labs	Oligomers
				Monomers
				Photoinitiators
				Stabilizer
Cosmos Bio Splint	3D printing resin	Transparent	Yller	Pigment
NightGuard Firm	3D printing resin	Pigmented	SprintRay	20–50% Proprietary methacrylate oligomer*
				20–50% Proprietary methacrylate monomer*
				0.3–5% proprietary photoinitiator*
				Proprietary pigment 1*
				Proprietary pigment 2*
Resina Auto	Self-polymerizing acrylic resin	Transparent	TDV Dental	Liquid phase: Methyl methacrylate
				Solid phase:
				0–100.00% Methyl methacrylate
				0–5.0% Methyl acrylate
				0–2.0% Dioctyl phthalate
				0–2.0% Benzoyl peroxide

*Denotes that the specific chemical identity and/or exact percentage (concentration) of composition has been withheld as a trade secret.

### Sample preparation

The dimensions were 6 × 4 mm (± 0.5) for Ra and KH and 25 × 2 × 2 mm (± 0.5) for FS and FM (n = 10). The 3D printing resins specimens were designed using Autodesk^®^ Meshmixer^TM^ v. 3.5.474 (Autodesk, Inc., San Rafael, USA) and imported into Chitubox^®^ Basic v1.9.4 (Chitubox, Guangdong, China). Four supports were added at an angle of 45° with a layer thickness of 50 μm per sample. Each resin was stirred for 3 min to ensure homogeneity before being poured into the printer tray following the recommendations of the manufacturer. Then, they were loaded into an Anycubic Photon S printer (Shenzhen Anycubic Technology Co., Guangdong, China) using liquid crystal display (LCD) technology and UV light at a wavelength of 405 nm and an energy of 50 W. After printing, the samples were washed for 5 min for PZ^21^ and 10 min for CS^22^ and NGF^23^ in 96.9% isopropyl alcohol. Subsequently, they were subjected to a 10-min post-cure process using a Cure Machine 2.0 Cure Machine 2.0 ultrasonic tank (Shenzhen Anycubic Technology Co., Guangdong, China).

CG was prepared using a stainless-steel mold (Odeme, Luzema, Brazil). The mold was positioned between two glass plates separated by polyester strips for better adaptation of the material and application of pressure to avoid bubbles and ensure a smooth surface. The disk samples were immersed in polystyrene resin (Darlene Handicrafts, Uberlândia, Brazil). The evaluation surface was inserted 2 mm deep into a utility wax sheet (Lysanda, São Paulo, Brazil) surrounded by a 25 × 10 × 10 cm cylindrical PVD tube and divided into two parts: the right side for initial tests (24 h) and the left side for tests after exposure (30 d). Once the resin covered the samples and polymerization was complete, the tube was removed, leaving the rest of the sample submerged in the resin. The polishing protocol was to use a sandpaper with particle sizes of 30 μm, 18 μm, and 15 μm with water in a rotary polishing machine (Arotec, São Paulo, Brazil). An extra-fine pumice stone (Iodontosul® Souza e Leonardi Ltda., Porto Alegre, Brazil) was applied, followed by White Spain high-gloss paste (Asfer, Indústria Química Ltda., São Caetano do Sul, Brazil), and a cotton polishing wheel (Clemara Cotton Brush No. 12 - Kota Knebel, Brazil) at 10,000 rpm and 1 N force for 1 min, which uses a polishing motor dental drill (Essence Dental VH, São Bernardo do Campo, Brazil). Finally, the samples were exposed to artificial saliva (S) (Kiropharma, Uberlíndia, Brazil), natural water (W), or 0.12% chlorhexidine (C) (Farmácia N. Sra. Guia, Uberlândia, Brazil), and Corega tabs (CTs) (GlaxoSmithKline Ltda., Rio de Janeiro, Brazil) for 30 d (Tables 2 and 3).

**Table 2:** Number of samples prepared and exposure time based on hours of sleep (8 h simulating exposure in saliva within the oral cavity, with the remaining 16 h dedicated to preservation). Before exposure - 24 h after manufacturing (T0)

Material	PZ	CS	NGF	CG	Total
Hours	10	10	10	10	40

**Table 3:** Number of samples prepared and exposure time based on hours of sleep (8 h simulating exposure in saliva within the oral cavity, with the remaining 16 h dedicated to preservation). After exposure (30 days).

Tested conditions			*Post-exposure storage conditions			Material			
Time per day	Preservation	Solution amount (ml)	Remaining time	Condition	Solution amount (ml)	PZ	CS	NGF	CG
8 h	S	5	16 h	Natural environment (dry)	_	10	10	10	10
16 h	W	5	8 h	S	5	10	10	10	10
16 h	C	5	8 h	S	5	10	10	10	10
5 min	CT	200	8 h	S	5	10	10	10	10
Total						160			

Post-exposure storage conditions for the groups natural water (W), chlorhexidine 0.12% (C), and Corega tabs (CTs) in artificial saliva (S), simulating sleep hours, were in an oven at 37

### Surface Roughness (Ra)

The Ra was measured using a portable surface roughness tester (SJ-412 50MM-4 MN, Mitutoyo, São Paulo, Brazil). The samples were fixed to glass plates using utility wax. The device has a diamond tip with a radius of 0.5 mm. It moved at speed of 0.25 mm/s and traversed 0.25 mm (cut-off length), measuring in micrometers (µm). Five analyses were performed (three horizontally and two vertically) after rotating the sample by 90° at a distance of 1 mm between them. Finally, a single representative value (Ra) was obtained as the average.

### Knoop microhardness (HK)

Five readings were obtained at a separation of 1 mm (three in the inner third and two in the middle) using a microhardness tester (FM-700, Future-Tech, Tokyo, Japan) at a load of 25 g for 10 s.

### Flexural strength (σ) and flexural modulus (E)

The samples were tested for three-point FS using a universal testing machine (Instron ElectroPulsTM E3000, Instron, High Wycombe, United Kingdom) controlled by the Bluehill Universal software (Instron Training Center, Norwood, USA) at a speed of 5 mm/min until the fracture occurred. The formula σ = 3FL / 2BH was used, where F, L, B, and H represent the maximum applied force (N), distance between support beams (20 mm), sample width (mm), and sample thickness (mm), respectively. The FM was determined using a deflectometer (W-E401-J, E-Series Deflectometer; Instron, Norwood, USA). The deflectometer was positioned at the center of the sample base during the test to measure the deflection when the load was applied following the formula E = FL^3^ / 4BH^3^d, where F, L, B, H, and d represent the maximum load (N), sample length (20 mm), sample width (mm), sample height (mm), and deflection (mm) corresponding to load F, respectively.

### Statistical analysis

Data were tabulated in a spreadsheet and imported into Jamovi statistical software Jamovi 2.3.26 (The Jamovi Project, Sydney, Australia). Three-way repeated measures ANOVA and Tukey Test were used for Ra and KH, while the Kruskal–Wallis test with multiple comparisons was employed for FS and FM, with a significance level of α = 0.05.

## Results

### Surface Roughness (Ra)

**Figure 1:**
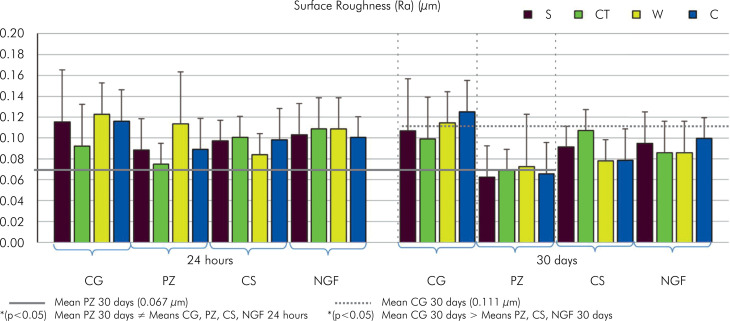
Means and standard deviations of surface roughness (Ra) (µm) in 24 h and 30 d.

In terms of time and material, Tukey’s test revealed no significant differences between the groups at 24 h. After 30 d, PZ showed significantly lower values than the other groups (p < 0.05). CG maintained its values; however, they were higher than those of the other groups (p < 0.05). In the material-preservation interaction, PZ and CS showed significantly lower values than CG under C, CS differed from CG in W, and PZ varied from CG in S, displaying low values (p < 0.05). The data are presented in Figure 1.

### Knoop microhardness (KH)

**Figure 2:**
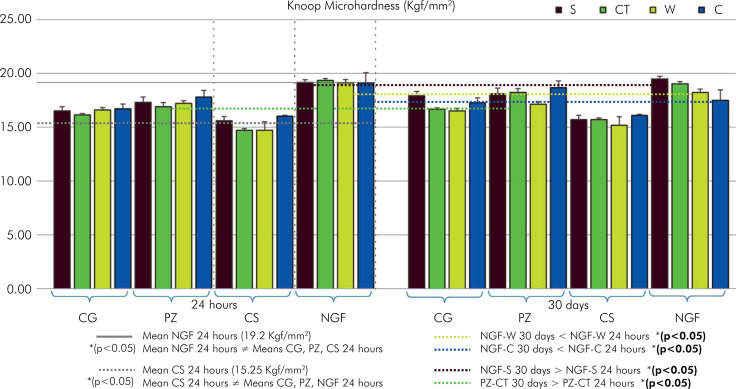
Means and standard deviations of Knoop microhardness (Kgf/mm2) in different preservations at 24 h and 30 d.

At 24 h (Figure 2), NGF exhibited significantly higher values than the other groups (p < 0.05). This was followed by PZ, which showed significant differences from CS but not from CG, while the CS group presented low values compared to those of the others. After 30 d, NGF decreased in W and C (p < 0.05). NGF and PZ exhibited elevated values in S and CT, respectively. The CG displayed elevated and distinct values of S compared to their counterparts at T0.

### Strength (σ) and flexural modulus (E)

**Figure 3:**
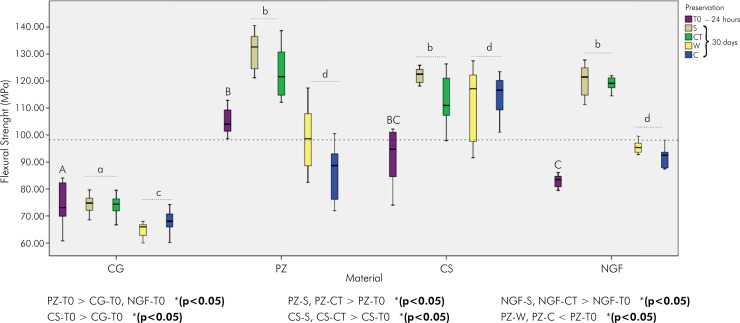
Medians and quartiles of flexural strength (MPa) in 24 h (T0) and 30 d.

In the assessment at T0 (Figure 3) for FS, PZ exhibited the highest and statistically significant value compared to CG and NGF (104.03 MPa; 73.11 MPa; 83.45 MPa, respectively) (p < 0.05). The CS (94.70 MPa) differed from that of the CG (p = 0.024). After 30 d, all 3D printing resins showed increased S and CT values compared with their respective T0 and CG values (p < 0.05). However, the PZ decreased in W (98.59 MPa) and C (88.71 MPa) (p < 0.05). The CG did not show any significant difference.

Different letters indicate significant differences – uppercase letters indicate comparisons between materials at T0 (24 h) and lowercase letters indicate comparisons between preservations (30 d) (p < 0.05).

**Figure 4:**
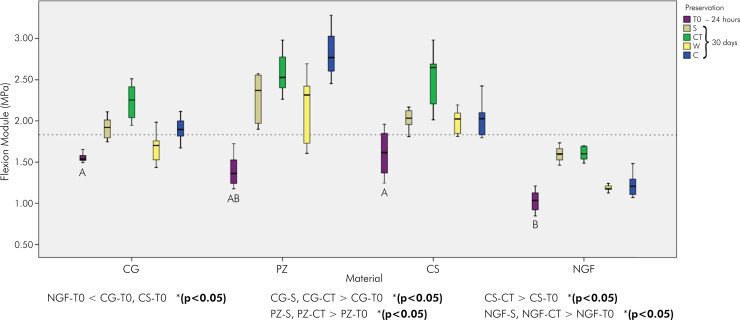
Medians and quartiles of flexion module (MPa) in 24 h (T0) and 30 d.

For the FM at T0 (Figure 4), NGF had the lowest and significant value compared to CS and CG (1.03 MPa; 1.61 MPa; 1.54 MPa, respectively) (p < 0.05). After 30 d, all materials exhibited high and significant values when subjected to CT and S compared to their equivalents at T0. NGF displayed the lowest overall values.

Different letters indicate significant differences – uppercase letters indicate comparisons between materials at T0 (24 h) and lowercase letters indicate comparisons between preservations (30 d) (p < 0.05).

## Discussion

The null hypothesis of this study was rejected. The ideal protocol for occlusal device hygiene can ensure adequate disinfection, nontoxicity, accessibility, and prevention of any alteration in their mechanical properties.^19^ However, this study revealed that preservation of the OS can affect their mechanical properties.

Commonly recommended techniques for disinfecting OS include immersion in 0.12% chlorhexidine gluconate or effervescent tablets.^24^ This study found that, after 30 d, PZ showed a significant decrease in Ra, particularly with C and W. Owing to its cationic structure and substantivity,^24^ chlorhexidine remained on the surface and released gradually. Managing the contact time with the disinfectant is crucial to avoid changes in the surface and color of the prosthesis.^25^ In this study, the contact time was 16 h for 30 d, which can explain the changes in the texture of the material.

The low Ra values in the PZ after 30 d in W highlighted significant differences over time and between the materials and preservation methods. Finishing and polishing are essential for reducing roughness; however, they depend on the instruments and protocols used.^26^ In this study, CG exhibited higher roughness, likely because of residual monomers causing pore formation and increased susceptibility to sorption.^27^ This solubility can lead to material loss and rougher surfaces.^28^

Effervescent tablets containing alkaline peroxides offer an alternative that does not require precise dosing. Their effectiveness comes from effervescence, which produces oxygen-free radicals with antimicrobial properties.^19^ Studies showed a minimal impact on the physical and mechanical properties of denture bases.^29-31^ However, high peroxide content in strongly alkaline solutions can damage prosthetic materials and rough surfaces.^22,26,32^ In this study, despite using water to dissolve CT, Ra increased in the CS and CG groups after 30 d compared to T0, although without statistical significance.

Layer thickness and post-curing can affect the surface quality of printed objects.^33^ The optimal thickness is between 25–100 µm. In this study, a 50 µm thickness was selected for improved light passage and compaction during polymerization. LCD printers produce smoother surfaces,^28,34^whereas digital light processing (DLP) printers are efficient because of their higher light intensity.^34,35^ PZ exhibited lower values after 30 d because of its compatibility with DLP, LCD, and SLA technologies. Printing at a 45° angle relative to the platform results in low roughness values and higher brightness after polishing,^15,36^ which explains the outcomes of this study, where all samples were printed at this angle.

KH measures the resistance of the material to permanent deformation.^10^ In 24 h, NGF displayed elevated and significant hardness; however, storage in the water can reduce the hardness of printed materials.^6,10^This explains the notable drop in values after 30 d of water immersion in this group. Further, hardness can be influenced by the curing method, UV light exposure time, or heat treatments.^6^ NGF exhibited the highest hardness values within the first 24 h. Considering the lack of specific post-curing guidance for this resin, a 10-min duration was selected, informed by the instructions for similar evaluated resins.

Therefore, elevated values within 24 h could be attributed to the resin composition.^10,37^ The “safety data sheet” provided by SprintRay on their website lists components and proportions as “trade secrets.”^32^ The resin includes methacrylic acid esters, photoinitiators, pigments, and additives. In contrast, the CS resin showed low hardness values after 24 h, containing oligomers, monomers, photoinitiators, stabilizers, and pigments, with specific percentages undisclosed.^20^ The 10-min post-curing may have been impacted by the monomer loading percentage.

The oxygen released by the effervescent tablet can cause the chemical softening of the surface of polyamide resins, damaging the polymer chains and compromising their hardness.^25^ In contrast, this study observed an increase in KH after 30 d for the PZ, CS, and CG groups, possibly because of their composition based on methacrylate, which differs from polyamide.

ISO 20795-1 requires a minimum FS of 65 MPa for prosthesis-based polymers, and the materials used in this study meet this standard.^9,18^Low viscosity is crucial for 3D printing resins. Increasing the resin temperature reduces viscosity, facilitating free radical and polymer chain movement. This leads to higher conversion rates, increased crosslinking and polymer swelling, limited water sorption, and improved material flexibility for bending.^38^

The use of CT increased the FS of PZ, CS, and NGF after 30 d. An in vitro study revealed a linear correlation between FS and solubility, thereby suggesting that water affects material aging by decomposing chemical bonds in the polymer network, which leads to values.^27^ However, in this study, the CT contact with the samples was limited to only 5 min per day in 200 ml of water for 30 d, indicating minimal immersion and no resulting decrease in values.

A FM indicates material stiffness, with high values indicating greater resistance to elastic deformation and uniform force distribution, which are advantageous in the clinical setting.^9,39,40^ NGF displayed the lowest FM at 24 h, which differs significantly from the other groups. After 30 d, NGF exhibited an increased modulus of flexion with S and CT exposure, possibly because of the environmental or chemical agents^40^ in these preservations reducing the resin water content, thus enhancing stiffness.

The limitations include reduced details on the resin composition, which hinder a comprehensive analysis of the material’s response, as well as environmental factors and chemicals. Furthermore, a 16-h exposure time to C was selected to standardize the solution change intervals. However, we acknowledge that this duration may be considered excessive because it matches the exposure time of W. In an in vitro study, clinical oral use was not replicated. Finally, future research should focus on post-curing effects, calibration of 3D printing resins, and patient-centered clinical evaluations.

## Conclusions

The preservation conditions significantly affect the mechanical properties of 3D printing resins used for OS manufacturing. All preservation conditions reduced the Ra of the PZ. Preservation in C and W tended to decrease the NGF hardness. The use of CT and preservation in S increased the FS of all 3D printing resins. Finally, CT increased the flexural moduli of both PZ and CS.
